# *Pfhrp2* and *pfhrp3* polymorphisms in *Plasmodium falciparum* isolates from Dakar, Senegal: impact on rapid malaria diagnostic tests

**DOI:** 10.1186/1475-2875-12-34

**Published:** 2013-01-24

**Authors:** Nathalie Wurtz, Bécaye Fall, Kim Bui, Aurélie Pascual, Mansour Fall, Cheikhou Camara, Bakary Diatta, Khadidiatou Ba Fall, Pape Saliou Mbaye, Yaya Diémé, Raymond Bercion, Boubacar Wade, Sébastien Briolant, Bruno Pradines

**Affiliations:** 1 Unité de Parasitologie, Département d’Infectiologie de Terrain, Institut de Recherche Biomédicale des Armées, Marseille, France; 2 Aix Marseille Université, Unité de Recherche sur les Maladies Infectieuses et Tropicales Emergentes, UM 63, CNRS 7278, IRD 198, Inserm 1095, Marseille, France; 3 Laboratoire d’étude de la chimiosensibilité du paludisme, Fédération des laboratoires, Hôpital Principal de Dakar, Dakar, Sénégal; 4 Centre National de référence du Paludisme, Marseille, France; 5 Service de réanimation médicale, Hôpital Principal de Dakar, Dakar, Sénégal; 6 Service des urgences, Hôpital Principal de Dakar, Dakar, Sénégal; 7 Service de pathologie infectieuse, Hôpital Principal de Dakar, Dakar, Sénégal; 8 Département de médecine interne et spécialités médicales de pathologie tropicale, Hôpital Principal de Dakar, Dakar, Sénégal; 9 Chefferie, Hôpital Principal de Dakar, Dakar, Sénégal

**Keywords:** Malaria, *Plasmodium falciparum*, Diagnosis, Rapid diagnostic test, *Plasmodium falciparum* histidine-rich protein 2, Anti-malarial

## Abstract

**Background:**

An accurate diagnosis is essential for the rapid and appropriate treatment of malaria. The accuracy of the histidine-rich protein 2 (PfHRP2)-based rapid diagnostic test (RDT) Palutop+4® was assessed here. One possible factor contributing to the failure to detect malaria by this test is the diversity of the parasite PfHRP2 antigens.

**Methods:**

PfHRP2 detection with the Palutop+4® RDT was carried out. The *pfhrp2* and *pfhrp3* genes were amplified and sequenced from 136 isolates of *Plasmodium falciparum* that were collected in Dakar, Senegal from 2009 to 2011. The DNA sequences were determined and statistical analyses of the variation observed between these two genes were conducted. The potential impact of PfHRP2 and PfHRP3 sequence variation on malaria diagnosis was examined.

**Results:**

Seven *P. falciparum* isolates (5.9% of the total isolates, regardless of the parasitaemia; 10.7% of the isolates with parasitaemia ≤0.005% or ≤250 parasites/μl) were undetected by the PfHRP2 Palutop+4® RDT. Low parasite density is not sufficient to explain the PfHRP2 detection failure. Three of these seven samples showed *pfhrp2* deletion (2.4%). The *pfhrp3* gene was deleted in 12.8%. Of the 122 PfHRP2 sequences, 120 unique sequences were identified. Of the 109 PfHRP3 sequences, 64 unique sequences were identified. Using the Baker’s regression model, at least 7.4% of the *P. falciparum* isolates in Dakar were likely to be undetected by PfHRP2 at a parasite density of ≤250 parasites/μl (slightly lower than the evaluated prevalence of 10.7%). This predictive prevalence increased significantly between 2009 and 2011 (*P* = 0.0046).

**Conclusion:**

In the present work, 10.7% of the isolates with a parasitaemia ≤0.005% (≤250 parasites/μl) were undetected by the PfHRP2 Palutop+4® RDT (7.4% by the predictive Baker’model). In addition, all of the parasites with *pfhrp2* deletion (2.4% of the total samples) and 2.1% of the parasites with parasitaemia >0.005% and presence of *pfhrp2* were not detected by PfHRP2 RDT. PfHRP2 is highly polymorphic in Senegal. Efforts should be made to more accurately determine the prevalence of non-sensitive parasites to pfHRP2.

## Background

The early and accurate diagnosis of malaria is important for the effective management and treatment of this disease to reduce morbidity and mortality. Malaria diagnosis relies on the microscopic examination of blood smears, which remains the standard method. The World Health Organization (WHO) recently recommended the adoption of universal testing to confirm the presence of malaria parasites prior to the use of artemisinin-based combination therapy (ACT). In cases where microscopic examination cannot be performed, rapid diagnostic tests (RDTs) are the best alternative for diagnosis. RDTs can be useful because they provide quick and accurate diagnoses, thereby leading to timely and accurate treatment and reducing the severity and economic burden of the disease. Today, many RDTs are commercially available, and they utilize immunochromatographic methods to detect parasite-specific antigens in lysed blood cells [1]. Most products detect *Plasmodium falciparum*-specific proteins and target either the *P. falciparum* histidine-rich protein 2 (PfHRP2) or the *P. falciparum* lactate dehydrogenase (PfLDH). Certain RDTs can detect both *P. falciparum*-specific and pan-specific antigens (aldolase (pALD) or pan-malaria (pLDH)) [1]. Many factors related either to the parasite or to the test may affect the performance of malaria RDTs. Although pALD and pLDH appear to be highly conserved [2,3], it has been reported that *pfhrp2* sequence variations, particularly with regard to certain amino acid repeats, can affect the sensitivity of HRP2-based RDTs [3-7]. These polymorphisms have been detected in the Asia-Pacific region [4,5], India [6], Madagascar [3] and in a clinical case in Uganda [7]. In addition, misdiagnosis may also arise from gene deletions that prevent the expression of proteins by the parasite. More recently, the deletions of *pfhrp2* and *pfhrp3* were reported as the causes of false-negative diagnoses in populations from Peru [8,9], Mali [10], India [11] and in a clinical case from Brazil [12].

The aims of this study were to investigate the genetic diversity of the *pfhrp2* and *pfhrp3* genes in *P. falciparum* clinical isolates from Dakar, Senegal, and its impact on predictive HRP2-based RDT diagnostic sensitivity using the Baker’s regression model [4]. Then, predictive results were compared to results obtained with use of the HRP2-based RDT Palutop+4® (All Diag, Strasbourg, France), microscopic examination and real-time polymerase chain reaction.

## Methods

### *Plasmodium falciparum* isolates

A total of 136 blood samples were selected for analysis from the Hôpital Principal de Dakar. The samples, stored at −20°C, were from patients who came to the hospital for a malaria consultation and were recruited for the evaluation of *P. falciparum ex vivo* susceptibility to anti-malarial drugs [13-15]. The samples represented three malaria seasons: 14 October 2009 to 19 January 2010, 20 July 2010 to 18 February 2011 and 3 October 2011 to 13 January 2012. Informed verbal consent was obtained from patients or their parents prior to blood collection. All the tests were performed on the same blood samples used for the malaria diagnosis. The study was reviewed and approved by the ethics committee of the Hôpital Principal de Dakar.

### Diagnosis of malaria

The diagnosis of malaria was assessed by microscopic examination, use of HRP2-based RDT and real-time polymerase chain reaction. At recruitment, thin blood smears were stained using a RAL®kit (Réactifs RAL, Paris, France) and were examined to determine the parasite density and diagnosis.

**Figure 1:**
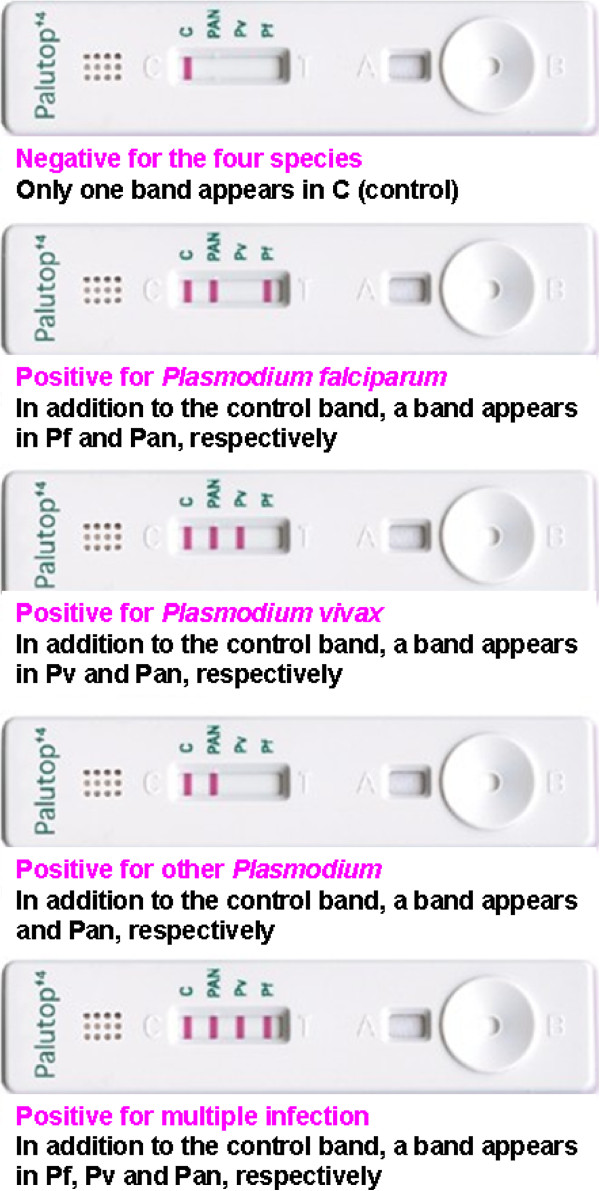
**Malaria diagnosis by either the Palutop+4® rapid diagnostic test, which recognizes the *Plasmodium falciparum*-specific histidine rich protein-2 (PfHRP2), the *Plasmodium vivax*-specific LDH test for *P. vivax *detection and the pan-malaria-specific pLDH test for the detection of *P. falciparum*, *P. vivax*, *Plasmodium ovale *or *Plasmodium malariae*.**

Immunochromatographic rapid tests (RDT Palutop+4®) were used to perform the diagnosis for four species of *Plasmodium*. Palutop+4® is a rapid test that uses whole blood for the detection of the *P. falciparum*-specific histidine rich protein-2 (PfHRP2), the *Plasmodium vivax*-specific lactate dehydrogenase (LDH) and the pan-malaria-specific pLDH for *P. falciparum*, *P. vivax*, *Plasmodium ovale* or *Plasmodium malariae* (Figure 1). 

To confirm the diagnosis obtained by the RDTs and the blood smear examination, *P. falciparum* and *P. vivax* detection was performed by real-time PCR using a LightCycler® (Roche, Meylan, France). The following oligonucleotide primers and probes, designed with Primer Express software v2.0 (Applied Biosystems), were used: forward 5’-TTTATGTATTGGTATAATTCGG-3’, reverse-5’- GGCAAATAACTTTATCATAGAATTGAC-3’ and probe-5’-FAM-TACACTACCAACACATGGGGCTACAAGAGGT-BBQ-3’ for the *P. falciparum* aquaglyceroporin gene (AJ413249); forward-5’-GTGGCCGCCTTTTTGCT-3’, reverse-5’-CCTCCCTGAAACAAGTCATCG-3’ and probe-5’-HEX-CATCTACGTGGACAACGGGCTCAACA-BHQ1-3’ for the *P. vivax* enoyl-acyl carrier protein reductase gene (AY423076) (Eurogentec, Angers, France). Each parasite species was detected separately. The individual real-time PCR amplifications were carried out using the previously described conditions [16].

### Nucleic acid extraction

Total genomic DNA of each strain was isolated using the QIAamp® DNA Mini kit according to the manufacturer’s recommendations (Qiagen, Germany).

### *pfhrp2* sequencing

A 905-nucleotide fragment of exon 2 of the *P. falciparum* histidine-rich protein 2 (*pfhrp2*) gene (M13986) was amplified by PCR using the *pfhrp2*-sense 5’- TGTGTAGCAAAAATGCAAAAGG -3’ and the *pfhrp2*-antisense 5’- TTAATGGCGTAGGCAATGTG -3’ primers [7]. The reaction mixture for the PCR amplification included 5 μl of genomic DNA, 2.5 μl of 10X reaction buffer (Eurogentec, Angers, France), 0.5 μM of each primer, 200 μM of a deoxynucleoside triphosphate mixture (dGTP, dATP, dTTP and dCTP) (Euromedex, Souffelweyersheim, France), 1.5 mM MgCl2 and 1 unit of RedGoldStar® DNA polymerase (Eurogentec, Angers, France) to a final volume of 25 μl. The thermal cycler (T3 Biometra, Archamps, France) was programmed as follows: 94°C for 5 min; 45 cycles of 94°C for 30 sec, 62°C for 40 sec and 72°C for 90 sec; 10 min extension step at 72°C. The PCR products were loaded on a 1% agarose gel containing 0.5 μg/mL ethidium bromide. The amplicons were purified using the High Pure 96 UF Cleanup Kit according to the manufacturer’s instructions (Roche). The purified fragments were sequenced using the BigDye Terminator v3.1 Cycle Sequencing Kit (Applied Biosystems) using the primers described above. The sequencing reaction products were purified using the BigDye XTerminator® Purification Kit (Applied Biosystems) in accordance with the manufacturer's instructions. The purified products were sequenced using an ABI Prism 3100 analyzer (Applied Biosystems). The sequences were analysed and translated using the Vector NTI advance (TM) software (version 11, Invitrogen, Cergy Pontoise, France). The amino acid repeats were identified by numerical code (1–18) as described by Baker *et al.*[5].

### *pfhrp3* sequencing

A 727-nucleotide fragment of exon 2 of the *P. falciparum* histidine-rich protein 3 (*pfhrp3*) gene (U69552) was amplified by PCR using the *pfhrp3*-sense 5’- TGTTTAGCAAAAATGCAAAAGGACT -3’ and *pfhrp3*-antisense 5’- TGTAAGTGATGCGTAGTGGCA -3’ primers (Eurogentec, Angers, France), which were designed with the NCBI/Primer-BLAST online tool. The reaction mixture for the PCR amplification included 2 μl of genomic DNA, 2.5 μl of 10X reaction buffer (Eurogentec, Angers, France), 0.5 μM of each primer, 200 μM of a deoxynucleoside triphosphate mixture (dGTP, dATP, dTTP and dCTP) (Euromedex, Souffelweyersheim, France), 1.5 mM MgCl2 and 1 unit of RedGoldStar® DNA polymerase (Eurogentec, Angers, France) in a final volume of 25 μl. The thermal cycler (T3 Biometra, Archamps, France) was programmed as follows: 94°C for 5 min; 40 cycles of 94°C for 30 sec, 54°C for 40 sec and 72°C for 50 sec; 10 min extension step at 72°C. The PCR products were loaded on a 1% agarose gel containing 0.5 μg/mL ethidium bromide. The amplicons were purified using the High Pure 96 UF Cleanup Kit according to the manufacturer’s instructions (Roche). The purified fragments were sequenced using the BigDye Terminator v3.1 Cycle Sequencing Kit (Applied Biosystems) using the primers described above. The sequencing reaction products were purified as described in the previous section.

### Assessment of *pfhrp2* and *pfhrp3* gene deletions

To confirm gene deletion and not negative PCR reactions due to poor quality of DNA, independent PCR experiments were assessed on samples with no amplicon detected by amplification of entire exon 2 of *pfhrp2* or *pfhrp3*, described in the previous sections. Further amplifications were performed across exon 1 and 2 of both *pfhrp2* and *pfhrp3* using primers previously described [8].

### Data analysis and statistical tests

The number of repeats were determined for the AHHAHHVAD pattern (type 1), AHHAHHAAD (type 2), AHHAHHAAY (type 3), AHH (type 4), AHHAHHASD (type 5), AHHATD (type 6), AHHAAD (type 7), AHHAAY type 8), AAY (type 9), AHHAAAHHATD (type 10), AHN (type 11), AHHAAAHHEAATH (type 12), AHHASD (type 13) and AHHAHHATD (type 14) for PfHRP2.

PfHRP2 sequences were classified into four groups as a function of the number of type 2 × type 7 repeats: group A PfHRP2 sequences (very sensitive) contained more than 100 type 2 × type 7 repeats, group B (sensitive) contained between 50–100 type 2 × type 7 repeats, group C (non-sensitive) contained < 43 repeats and "borderline" group I contained between 44 and 49 repeats.

The difference in the percentage of isolates belonging to group C for each malaria season and of detected or undetected parasites was analysed by the Chi-square test. The parasitaemia was classified into two groups: parasitaemia ≤0.005% and >0.005%. The association between the absence of a diagnosis by PfHRP2 and the particular group of HRP2 sensitivity, parasitaemia or type of amino acid repeat was analysed using the Wilcoxon rank sum test and the Kruskal-Wallis rank sum test.

## Results

### *Plasmodium falciparum* diagnoses

The parasitaemia ranged from 0.0000 to 15.8% (median = 0.1%, Q1 = 0.02%; Q3 = 0.5%). 22.8% of the samples showed a parasitaemia ≤0.005% (≤250 parasites/μL).

#### *Plasmodium falciparum* false-negative diagnoses by the PfHRP2-based Palutop+4®

Of 118 isolates, regardless of parasite density, seven samples were not detected by the PfHRP2-based Palutop+4®. Three showed a parasitaemia ≤0.005% (≤250 parasites/μl). There was no significant difference in term of sample numbers (p = 0.246, Chi-square test) or in term of parasitaemia between detected (mean = 0.45%; standard deviation = 1.16%) and undetected samples (mean = 0.22%; standard deviation = 0.26%) (p = 0.595, Wilcoxon rank sum test). However, one of these samples was detected by the pan-malaria-specific pLDH and the *P. vivax*-specific LDH (Table 1). All of the seven samples were detected as *P. falciparum* by real-time polymerase chain reaction. 

**Table 1:** ***Plasmodium falciparum *false-negative diagnoses by the HRP2-based test**

	**Palutop+4®**			**Microscopy**		**RT-PCR**		**HRP2**^**a**^	**HRP3**^**b**^
Identification	Pan	Pf	Pv	Diagnostic	Parasitaemia	Pf	Pv		
186/1	+	−	+	Pf	0.5%	+	−	−	−
212/1	−	−	−	Pf	0.02%	+	−	+ (B)	−
55/2	−	−	−	Pf	0.5%	+	−	+ (B)	−
56/3	−	−	−	Pf	0.5%	+	−	−	+
150/3	−	−	−	Pf	0.001%	+	−	−	−
157/3	−	−	−	−^c^		+	−	+ (B)	−
183/3	−	−	−	Pf	0.004%	+	−	+ (C)	−

Pf = *Plasmodium falciparum*; Pv = *Plasmodium vivax*; RT-PCR = real-time polymerase chain reaction.

^a^ − If deletion of *pfhrp2* and + if presence of *pfhrp2.*

(B) Group B, sensitive to PfHRP2, detection, number of type 2 repeats × type 7 repeats between 50 to 100 repeats.

(C) Group C, non sensitive PfHRP2 detection, number of type 2 repeats × type 7 repeats < 43 repeats.

^b^ − If deletion of *pfhrp3* and + if presence of *pfhrp3.*

^c^ Thin and thick blood smears were negative.

Of the isolates detected as *P. falciparum* by PfHRP2-based Palutop+4®, 7.1% were not detected by the pan-malaria-specific pLDH. The parasitaemia ranged from 0.0000% to 0.5% (mean = 0.18%; standard deviation = 0.24%) for the undetected group and 0.0000% to 15.8% (mean = 0.48%; standard deviation = 1.20%) for the group detected by the pan-malaria-specific pLDH. There was no significantly difference in term of parasitaemia between the two groups (p = 0.144, Wilcoxon rank sum test).

#### *Plasmodium vivax* false-positive diagnoses the PfHRP2-based Palutop+4®

Five of 118 samples were detected as *P. vivax* by the RDT Palutop+4® (Table 2). These five samples were suspected as *P. falciparum* by microscopy and were confirmed as *P. falciparum* by real-time polymerase chain reaction. None of these samples was confirmed as *P. vivax*. These five samples showed a parasitaemia ≥0.005%. 

**Table 2:** ***Plasmodium vivax *false-positive diagnoses**

	**Palutop+4®**			**Microscopy**		**RT-PCR**	
Identification	Pan	Pf	Pv	Diagnostic	Parasitaemia	Pf	Pv
146/1	−	+	+	Pf	0.8%	+	−
186/1	+	−	+	Pf	0.5%	+	−
81/3	+	+	+	Pf	1.4%	+	−
113/3	+	+	+	Pf	2.0%	+	−
146/3	+	+	+	Pf	0.06%	+	−

Pf = *Plasmodium falciparum*; Pv = *Plasmodium vivax*; RT-PCR = real-time polymerase chain reaction.

### Genetic diversity of *Plasmodium falciparum* HRP2 and HRP3

For PfHRP2, 122 PCR fragments were successfully amplified. No amplicon was detected for three samples (186/1, 56/3 and 150/3) by the two methods. The *pfhrp2* gene was deleted in these three samples (2.4% of the samples). Thirteen of the 14 previously identified amino acid repeats were detected [4] (Table 3). Of the 122 PfHRP2 sequences, 120 unique sequences were identified. Only three PfHRP2 sequences did not begin with the type 1 repeat (AHHAHHVAD). The type 2 repeat (AHHAHHAAD) was observed in 100% of the sequenced isolates, the type 3 (AHHAHHAAY) in 89%, the type 5 (AHHAHHASD) in 75%, the type 6 (AHHATD) in 98%, the type 7 (AHHAAD) in 99%, the type 8 (AHHAAY) in 84% and the type 10 (AHHAAAHHATD) in 75%. Several of the repeats occurred in only a few isolates: the type 4 (AHH) in 20 isolates (16%), the type 9 (AAY) in two isolates, the type 12 (AHHAAAHHEAATH) in only one isolate, the type 13 (AHHASD) in nine isolates and the type 14 (AHHAHHATD) in five isolates. The type 11 repeat (AHN) was not detected in *P. falciparum* parasites from Dakar. 

**Table 3:** **The distribution of amino acid repeats (minimum to maximum number) in PfHRP2 and PfHRP3 from *Plasmodium falciparum *isolates from Dakar, Senegal**

**Code**	**Amino-acid repeats**	**PfHRP2**	**PfHRP3**
1	AHHAHHVAD	0–8	0–4
2	AHHAHHAAD	4–17	0
3	AHHAHHAAY	0–3	0
4	AHH	0–3	1
5	AHHAHHASD	0–2	0
6	AHHATD	0–7	0
7	AHHAAD	0–14	1–2
8	AHHAAY	0–2	0
9	AAY	0–1	0
10	AHHAAAHHATD	0–3	0
11	AHN	0	0
12	AHHAAAHHEAATH	0–1	0
13	AHHASD	0–2	0
14	AHHAHHATD	0–1	0
15	AHHAHHAAN	0	0–1
16	AHHAAN	0	3–19
17	AHHDG	0	3–9
18	AHHDD	0	0–6

The PfHRP2 sequences were classified as described in the Methods section. The results of the classification of the PfHRP2 sequences into type 2 × type 7 repeat groups are shown in Table 4. 

**Table 4:** The distribution of PfHRP2 sequences (number, no; percentage, %) into four groups based on the number of type 2 × type 7 repeats: group A (very sensitive) PfHRP2 sequences contained more than 100 type 2 × type 7 repeats, group B (sensitive) contained between 50 to 100 type 2 × type 7 repeats, group C (non-sensitive) contained <43 repeats and the "borderline" group I contained between 44 and 49 repeats

	**Group A**		**Group B**		**Group I**		**Group C**	
Year	no	%	no	%	no	%	no	%
2009–2010	9	23.1	30	76.9	0	0	0	0
2010–2011	7	17.5	29	72.5	2	5	2	5
2011–2012	5	11.6	28	65.1	3	7.0	7	16.3
**2009–2012**	**21**	**17.2**	**87**	**71.3**	**5**	**4.1**	**9**	**7.4**

The number of isolates belonging to group C (non-sensitive to the HRP2 test) increased significantly between 2009 and 2011 (*P* = 0.0046, Chi-square test).

For PfHRP3, 109 PCR fragments were successfully amplified. No amplicon was detected for 16 samples by the two methods. The *pfhrp3* gene was deleted in 12.8% of the samples. Seven of the eight previously identified amino acid repeats were detected [4] (Table 3). Of the 109 PfHRP3 sequences, 64 unique sequences were identified. The type 1 repeat (AHHAHHAAD) was observed in 99% of the sequenced isolates, the type 4 (AHH) in 100%, the type 7 (AHHAAD) in 100%, the type 15 (AHHAHHAAN) in 99%, the type 16 (AHHAAN) in 100%, the type 17 (AHHDG) in 100% and the type 18 (AHHDD) in 99%. The type 2 repeat (AHHAHHAAD) was not detected in *P. falciparum* parasites from Dakar.

### Comparison between the HRP2-based RDT results and genetic polymorphisms or expected frequency determined by the Baker’s regression model

The three samples (186/1, 56/3 and 150/3) with deletion of *pfhrp2* (2.4%) were not detected by the PfHRP2-based Palutop+4®. One of these three samples (186/1) was detected by the pan-malaria-specific pLDH. Of the 16 samples with deletion of *pfhrp3* (12.8%), six were not detected by the PfHRP2-based Palutop+4®.

Of the four samples not detected by the PfHRP2-based Palutop+4® but with PfHRP2, only one (183/3) was classified in group C (non-sensitive) according to the Baker’s regression model. In addition, of the nine samples expected to be not detected by PfHRP2-based RDT, the sample 183/3 was the only one which was not detected by the PfHRP2-based Palutop+4®. There was no significant difference in parasitaemia between the four predict groups (p = 0.676, Kruskal-Wallis rank sum test). However, there was a significant association between the absence of a diagnosis by PfHRP2 RDT and the number of type 2 repeats (AHHAHHAAD) (*P* = 0.0463).

However, there was a significant association between the absence of a diagnosis by PfHRP2 RDT and the number of type 2 repeats (AHHAHHAAD) (*P* = 0.0463). There was no significant association between the absence of a diagnosis by PfHRP2-based Palutop+4® and the genetic diversity of other PfHRP2 repeats: type 1 (AHHAHHVAD) (*P* = 0.4634, Fisher’s Exact test), type 3 (AHHAHHAAY) (*P* = 0.7852), type 4 (AHH) (*P* = 1), type 5 (AHHAHHASD) (*P* = 0.7589), type 6 (AHHATD) (*P* = 0.1959), type 7 (AHHAAD) (*P* = 0.1872), type 8 (AHHAAY) (*P* = 1), type 9 (AAY) (*P* = 1), type 10 (AHHAAAHHATD) (*P* = 0.8402), type 12 (AHHAAAHHEAATH) (*P* = 1), type 13 (AHHASD) (*P* = 1) and type 14 (AHHAHHATD) (*P* = 1).

Among the 109 sequences of HRP3, only one isolate was not detected by PfHRP2-based Palutop+4®. There was no association between the absence of a diagnosis by PfHRP2-based Palutop+4® and the genetic diversity of PfHRP3.

## Discussion

The WHO recently recommended adopting universal testing to confirm the presence of malaria parasites prior to the use of ACT, and in the cases where microscopic examination cannot be performed, the RDT would be the best alternative for confirmation. In 2006, the Senegalese National Malaria Control Programme recommended ACT as the first-line treatment for uncomplicated malaria and, in 2007, mandated testing for all suspected cases of malaria with *P. falciparum* using the HRP2-based RDT. PfHRP2-based RDTs are now commonly used with high adherence in Senegal [17-19]. These RDTs can provide a quick and accurate diagnosis, thereby leading to timely and appropriate malaria treatment and a reduction in the severity and economic burden of the disease.

In the present work, 5.9% of the total (n = 7) *P. falciparum* isolates and 10.7% of the isolates with parasitaemia ≤0.005% (≤250 parasites/μl) were undetected by the PfHRP2 Palutop+4® RDT. Moreover, of the isolates detected as *P. falciparum* by PfHRP2, 7.1% were not detected by the pan-malaria-specific pLDH. Five *P. falciparum* isolates (4.2%) were misdiagnosed as *P. vivax* parasites by the *P. vivax* LDH.

Three possible factors can affect the sensitivity of the PfHRP2-based RDT: parasite density, *pfhrp2* deletion and *pfhrp2* polymorphisms. The parasite density can not explain the failure of the detection by the PfHRP2 Palutop+4® RDT. There was no significant difference in term of sample numbers (p = 0.246, Chi-square test) or in term of parasitaemia between detected (mean = 0.45%; standard deviation = 1.16%) and undetected samples by HRP2 (mean = 0.22%; standard deviation = 0.26%) (p = 0.595, Wilcoxon rank sum test). In addition, the non-detection by the pan-malaria-specific pLDH was not associated with parasite density. There was no significantly difference in term of parasitaemia between the two groups (p = 0.144, Wilcoxon rank sum test). For the five *P. falciparum* isolates (4.2%) misdiagnosed as *P. vivax* parasites by the *P. vivax* LDH, all of these showed parasitaemia ≥0.005% (≥250 parasites/μl).

Another possible factor affecting the sensitivity of the PfHRP2-based RDT is the failure of the parasite to express the antigen, either due to genetic deletions, frame shift mutations or alterations in protein expression. The deletions of *pfhrp2* and *pfhrp3* were reported as the cause of false-negative diagnoses in populations from Peru [8,9], Mali [10], India [11] and in a clinical case from Brazil [12]. In addition, the levels of *pfhrp2* transcription and PfHRP2 protein expression varied between *P. falciparum* strains, and this may impact the detection sensitivity of the PfHRP2-based RDT [20]. The three samples with deletion of *pfhrp2* (2.4%) were not detected by the PfHRP2-based Palutop+4®. The deletion of *pfhrp2* is one of the factors of false-negative diagnoses using PfHRP2-based RDT. Of the 16 samples with deletion of *pfhrp3* (12.8%), six were not detected by the PfHRP2-based Palutop+4®. The frequency of *pfhrp2* deletion observed in Senegal is comparable to those estimated in Africa, like in Mali (2%) [10] or in India (4.2%) [11] and lower than those estimated in South America like in Peru (25.7 and 41%) [8,9]. As previously described in Peru [8], the proportion of parasites lacking *pfhrp3* is higher than those lacking *pfhrp2* in samples collected in Dakar. This suggests that parasites lacking *pfhrp3* may have been present in Senegal longer than those lacking *pfhrp2*.

Polymorphisms in the *pfhrp2* gene that can affect the sensitivity of PfHRP2-based RDTs have been detected in the Asia-Pacific region [4,5], India [3], Madagascar [3] and in a clinical case in Uganda [7]. Prior to this report, no data were yet available on the sequence variation of HRP2 from *P. falciparum* parasites from Senegal. Consistent with previous reports [5-7], the PfHRP2 was highly diverse in parasite isolates from Dakar. Of the 122 PfHRP2 sequences, 120 unique sequences were identified. In previous works, all *pfhrp2* sequences have begun with a type 1 repeat (AHHAHHVAD) [5-7]. However, three of the Senegalese isolate sequences in this study did not begin with a type 1 repeat. The type 11 repeat (AHN) was not detected in *P. falciparum* parasites from Dakar. This is consistent with isolates from Africa [4], except for one isolate from Cameroon [5] and one from northern Madagascar [3]. However, the Senegalese PfHRP2 sequences had certain characteristics: only one isolate possessed a type 12 repeat (AHHAAAHHEAATH), while the PfHRP2 from all the previously described isolates from Africa, the Pacific, South America or Asia contained one type 12 repeat [3,5]. The type 9 repeat has not been described in Africa [3-5]. Interestingly, two isolates from Dakar showed a type 9 repeat. Types 13 and 14 have rarely been observed in Africa [3-5], yet these two types of repeats were found in nine (7.4%) and five (4.1%) isolates from Dakar, respectively.

Finally, using the Baker’s regression model, it was shown that at least 7.4% of *P. falciparum* isolates in Dakar (group C) were likely to be undetected by PfHRP2-based RDT at a parasite density of ≤250 parasites/μl. In Madagascar, 9% of the isolates at a parasite density of ≤250 parasites/μl were predicted to be undetected by PfHRP2-based RDT. The number of isolates predicted to be non-sensitive to the PfHRP2-based RDT increased significantly between 2009 and 2011 (0% in 2009, 5% in 2010 and 16.3% in 2011; *P* = 0.0046). One hypothesis to explain this increase is the selection of parasites which contain less than 43 repeats (type 2 × type 7 repeats). PfHRP2-based RDTs are now commonly used with high adherence in Senegal [17-19]. Patients with negative PfHRP2-based RDT are not treated for malaria. If the parasites are undetected by the PfHRP2-based RDT, the delay of diagnose before treatment might permit an increase in the time required for development of sexual stages (gametocytes) and in their transmission to mosquitoes during a blood meal compared to parasites treated quickly. The transmission of parasites undetected by the PfHRP2-based RDT might be more important, leading to faster dissemination of these genotypes. The frequency of these genotypes could increase in coming years. This evolution should continue to be monitored in Senegal.

In the Dakar samples with low parasite density, the predicted prevalence was slightly lower than the evaluated prevalence (7.4% *versus* 10.7%). The predictive regression model developed by Baker cannot explain all of the PfHRP2 non-detection in Senegal. Of the four samples not detected by the PfHRP2-based Palutop+4® but with PfHRP2, only one (183/3) was classified in group C (non-sensitive) according to the Baker’s regression model. In addition, of the nine samples expected to be not detected by PfHRP2-based RDT, the sample 183/3 was the only one which was not detected by the PfHRP2-based Palutop+4®. However, this sample was the only one with parasitaemia ≤0.005% (≤250 parasites/μl).

It has been reported that, due to PfHRP3 and PfHRP2 structural homology, PfHRP3 can cross-react with HRP2-coated antibodies in the RDT. PfHRP3 has also contributed to the detection of *P. falciparum* malaria infections [21]. In this study, the genetic diversity of *pfhrp3* (64 different sequences) was less than that of *pfhrp2* (120 different sequences). All of the previously described repeat types, other than type 2, were present (99% to 100%). The type 2 repeat has never been described in HRP3 in Africa [3,4]. The Senegalese HRP3 sequences had characteristics in common with those of other African isolates [3,4] and were different from Indian isolates in that they did not contain type 1 repeats [6]. The most common repeat types between PfHRP2 and PfHRP3 in Africa are types 1 and 7. These shared repeats, particularly type 7, which is the only type described both in Africa and India, are likely the basis for the observed cross-reactivity between PfHRP3- and PfHRP2-specific monoclonal antibodies [21,22]. In addition, the type 7 repeat is one of the two selected types in the Baker’s regression model to predict non-detection of parasites by PfHRP2 at a density of ≤250 parasites/μl.

## Conclusion

In the present work, 10.7% of the isolates with a parasitaemia ≤0.005% (≤250 parasites/μl ) were undetected by the PfHRP2 Palutop+4® RDT (7.4% by the predictive Baker’model). In addition, all of the parasites with *pfhrp2* deletion (2.4% of the total samples) and 2.1% of the parasites with parasitaemia >0.005% and presence of *pfhrp2* were not detected by PfHRP2 Palutop+4® RDT. PfHRP2 (and PfHRP3), the most common target of malaria RDTs, is highly polymorphic in Senegal. Future efforts should be made to more accurately determine the prevalence of parasites that are non-sensitive to HRP2-detection-based assays. Better understanding of the structure of PfHRP2 and its diversity at the polymorphism and protein levels will contribute to the evaluation and improvement of malaria RDTs.

## Competing interests

The authors declare that they have no competing interests.

## Authors’ contributions

NW, KB and AP carried out the diagnostic tests and the molecular genetic studies. BF, MF, CC, BD, KBF, PSM, YD, RB and BW supervised, carried out and coordinated the field collections of patient isolates. NW, SB and BP conceived and coordinated the study. SB and BP analysed the data. NW, AP, SB and BP drafted the manuscript. All the authors read and approved the final manuscript.
